# The Phylogeography of MERS-CoV in Hospital Outbreak-Associated Cases Compared to Sporadic Cases in Saudi Arabia

**DOI:** 10.3390/v12050540

**Published:** 2020-05-14

**Authors:** Xin Chen, Dillon Charles Adam, Abrar Ahmad Chughtai, Sacha Stelzer-Braid, Matthew Scotch, Chandini Raina MacIntyre

**Affiliations:** 1 Biosecurity Research Program, Kirby Institute, Faculty of Medicine, University of New South Wales, Sydney, NSW 2052, Australia; d.c.adam@protonmail.com (D.C.A.); rainam@protonmail.com (C.R.M.); 2 School of Public Health and Community Medicine, Faculty of Medicine, University of New South Wales, Sydney, NSW 2052, Australia; abrar.chughtai@protonmail.com; 3 School of Medical Sciences, Faculty of Medicine, University of New South Wales, Sydney, NSW 2052, Australia; s.stelzer-braid@unsw.edu.au; 4 Virology Research Laboratory, Prince of Wales Hospital, Sydney, NSW 2031, Australia; 5 Biodesign Center for Environmental Health Engineering, Biodesign Institute, Arizona State University, Tempe, AZ 85287, USA; matthew.scotch@asu.edu; 6 College of Health Solutions, Arizona State University, Phoenix, AZ 85004, USA; 7 College of Public Service and Community Solutions, Arizona State University, Tempe, AZ 85004, USA

**Keywords:** MERS-CoV, phylogenetics, phylogeography, epidemiology, hospital outbreaks, nosocomial

## Abstract

This study compared the phylogeography of MERS-CoV between hospital outbreak-associated cases and sporadic cases in Saudi Arabia. We collected complete genome sequences from human samples in Saudi Arabia and data on the multiple risk factors of human MERS-CoV in Saudi Arabia reported from 2012 to 2018. By matching each sequence to human cases, we identified isolates as hospital outbreak-associated cases or sporadic cases. We used Bayesian phylogenetic methods including temporal, discrete trait analysis and phylogeography to uncover transmission routes of MERS-CoV isolates between hospital outbreaks and sporadic cases. Of the 120 sequences collected between 19 June 2012 and 23 January 2017, there were 64 isolates from hospital outbreak-associated cases and 56 from sporadic cases. Overall, MERS-CoV is fast evolving at 7.43 × 10^−4^ substitutions per site per year. Isolates from hospital outbreaks showed unusually fast evolutionary speed in a shorter time-frame than sporadic cases. Multiple introductions of different MERS-CoV strains occurred in three separate hospital outbreaks. MERS-CoV appears to be mutating in humans. The impact of mutations on viruses transmissibility in humans is unknown.

## 1. Introduction

MERS-CoV is a positive-sense, single-stranded RNA virus [1]. It is the first known lineage C beta-coronavirus with high pathogenicity in humans [2]. The virus was initially isolated from an infected human case in Bisha, Saudi Arabia in 2012 [3]. The MERS-CoV genome is classified into two clades, clade A and clade B. The complete genome of MERS-CoV ranges between 25 and 32 kb, encoding 15 to 16 non-structural proteins cleaved from two large open reading frames (ORF1a, ORF1b) and four structural proteins, including the envelope, membrane, nucleocapsid and spike proteins [1]. MERS-CoV was evolving at an average rate of 1.12 × 10^−3^ substitutions per site per year (95% credible interval (CI), (8.76 × 10^−4^, 1.37 × 10^−3^)) when 42 MERS-CoV genomes sampled between 2012 and 2013 were used for the estimation of evolutionary rate [4], showing around 40-fold faster evolution than severe acute respiratory syndrome (SARS) [5] and 1000-fold faster evolution than the influenza A and B viruses [6].

The exact source of MERS-CoV and virus transmission to human beings is still unknown. Camels and bats are suggested as possible reservoir hosts, after MERS-CoV genome sequences and ORFs were isolated from them [7]. However, epidemiological studies show that around 60% of human cases in Saudi Arabia have no identified animal or human case exposure [8]. Less than 40% of cases have an identified risk factor, and camel exposure has only been identified in 68 of 1186 cases in Saudi Arabia by the end of 2015 [8]. 

The epidemiology of MERS-CoV has been puzzling, with some nosocomial outbreaks which exhibit a classic epidemic pattern but most cases (at least 66%) are sporadic [8,9]. Of the sporadic cases with a known contact history, the majority have exposure to camels or confirmed human cases in the community [8]. In contrast, outbreak cases are predominantly healthcare-associated, and the reasons contributing to hospital outbreaks are complex, including inadequate triage, delayed diagnosis and inadequate control measures [10]. Based on a systematic review in 2019, there have been eight major hospital outbreaks in Saudi Arabia [10] since MERS-CoV was first reported in 2012, including: (i) Al Ahsa outbreak 2013 [11], (ii) Al-Madinah Al-Munawarrah outbreak 2013 [12], (iii) Prince Sultan Military Medical City outbreak 2014 [13], (iv) Jeddah outbreak 2014 [14], (v) Taif outbreak 2013–2014 [15] and (vi) three Riyadh outbreaks in 2015 [16], 2016 [10] and 2017 [10]. The large hospital outbreak reported in Al Ahsa in 2013 involved 23 human cases with 15 fatalities, and was epidemiologically assumed by person-to-person transmission [11]. Unusually, subsequent phylogenetic analysis of four isolates revealed the possibility of multiple independent introductions of MERS-CoV from the community [11], and more than five of 13 transmissions in this hospital outbreak could not be explained by person-to-person transmission [17]. Phylogeographic analysis using a larger size of MERS-CoV genome sequence could provide further understanding of the transmission and evolution of MERS-CoV.

To date, no studies have examined the different phylogenetic patterns or geographic distributions of MERS-CoV between hospital-associated and sporadic cases. Therefore, we aimed to compare the phylogeography of MERS-CoV between hospital outbreak-associated cases and sporadic cases in Saudi Arabia using Bayesian phylogenetic methods [18].

## 2. Materials and Methods

### 2.1. Data Collection and Data Linkage

#### 2.1.1. MERS-CoV Human Cases Epidemiological Dataset

We collected the publicly available data on the human cases of MERS-CoV infection reported from 20 September 2012 to 31 December 2018 in Saudi Arabia. Demographic data (age, gender, occupation) and location was sourced from the “Case List of Saudi Ministry of Health (MoH)/World Health Organization (WHO) Novel Coronavirus MERS Announced Cases” on FluTrackers [19]. We checked the linked original reports from the WHO MERS-CoV disease outbreak news [20] and the Saudi MoH [21] for each human case listed on FluTrackers [19] and extracted data on disease risk factors, including date of notification, date of symptoms onset, date of hospitalization, laboratory confirmation date, complications, death and type of case based on the specified contact history (hospital outbreak-associated case and sporadic case: camel-linked, sheep-linked, community-linked, unknown exposure) to enhance the dataset. We excluded cases where data was unavailable on all risk factors.

#### 2.1.2. MERS-CoV Complete Genome Sequences Dataset

We searched for all available MERS-CoV complete genome sequences from humans in Saudi Arabia through GenBank. We downloaded a total of 131 MERS-CoV sequences using Geneious v.11.0.4 [22], of which we removed 10 sequences that were not full-length. We included the remaining 120 complete genome sequences in our dataset. Each sequence contained the basic information of accession number, date of sample collection and location (city).

#### 2.1.3. Matching Sequences with Human Cases

We used the PubMed link in each GenBank record to identify the original papers of isolates. Here, we found additional data on risk factors that was used as criteria for the matching process. We classified all the criteria into three groups (Figure S1):

1. Demographic characteristics: age, gender, healthcare worker, contact history;
2. Location: county or town, city, province, region;
3. Date: collection date of isolate, lab confirmation date (0–10 days after collection date), symptoms onset date (14 days before collection date), death date (after collection date).

Based on the available information in the two datasets, we matched each sequence with the human case. Three levels of likelihood of matching were defined (Tables S2 and S3):

1. Unique isolates had at least one criterion from group 1 (and) group 2 (and) group 3 matched with a human case;
2. Likely unique isolates had at least one criterion from group 2 (and) 3 matched with a human case;
3. Possible unique isolates had at least one criterion from group 2 (or) 3 matched with a human case.

### 2.2. Compilation of MERS-CoV Genetic Sequences

Using Geneious v.11.0.4 [22], we annotated all the complete genome sequences with accession numbers. Setting NC_019843 as the RefSeq (length: 29,529 nts) [23], we assembled all 120 sequences and manually trimmed the 5’ and 3’ edges. We aligned the trimmed sequences using MAFFT v7.308 [24]. 

### 2.3. Comparison of the Phylogeography between Hospital Outbreak-Associated Cases and Sporadic Cases

#### 2.3.1. Temporal Analysis

We generated a maximum likelihood tree using RAxML v8.2 [25] and then visualized a root-to-tip regression of genetic distance against sampling time in Temporal Signal Estimation Tool (TempEst) v1.5.1 [26]. Based on the collection dates of isolates, we designed a total of 16 preliminary models in BEAUTi v1.10.2 [27] to determine the substitution models (Hasegawa–Kishino–Yano (HKY) or general-time-reversible (GTR)), molecular clock (relaxed or strict) and tree prior (constant, exponential, skygrid or skyline). We specified a Markov chain Monte Carlo (MCMC) length of 30 million with a sampling frequency of every 3000 steps. We implemented the simulations in BEAST [27] and performed marginal likelihood estimation (MLE), using path sampling (PS) and stepping-stone sampling (SS) to compare these models [28,29]. 

#### 2.3.2. Discrete Trait Analysis

We specified an asymmetric discrete trait phylogeographic model utilizing Bayesian Stochastic Search Variable Selection (BSSVS) to compare the geographic signals between “city” of host location and “type” (hospital outbreak-associated cases or sporadic cases). Based on the 16 preliminary models of best relative fit (Table S4), the final model specified a GTR model (Dirichelet prior for GTR rates and frequencies) under a relaxed molecular clock model (lognormal continuous time Markov Chains (CTMC) rate [30]) and a constant coalescent tree prior (uniform distribution) in BEAUTi v1.10.2 [27]. Rates between discrete traits were specified using a gamma distribution and non-zero rates a Poisson distribution. We specified the MCMC chain of 50 million, sub-sampled every 5000 steps and ran two rounds independently using BEAST v1.10.2 [27] to check any bias in parameter sampling space. Using Tracer v1.6 [31], we inspected the convergence of the generated log files with 10% burn-in removed. Combining tree files generated from two rounds by running LogCombiner in the BEAST package [27] (resampling 10,000 states at a lower frequency), we got nine thousand trees. Then we used Tree Annotator v1.10.2 [27] to construct a maximum clade credibility (MCC) tree and visualized the tree in Figtree v1.4.3 [32]. For each trait, we examined the association between trait and distribution on phylogeny using BaTs [33]. Complete model specification including prior parameter distribution specification is included in the supplementary file available at https://figshare.com/s/64f8f4dbdbcaaa4a8af4.

#### 2.3.3. Mapping the Transmission Routes 

Using SpreaD3 [34], we calculated the BF of significant non-zero rates between discrete locations and mapped transmission routes. We used a threshold of BF > 3 as sufficient support and adjusted routes in Adobe Illustrator CC 2019 [35] for visualization.

## 3. Results

### 3.1. Data Collection and Data Linkage

A total of 1791 human cases of MERS-CoV infection in Saudi Arabia were reported from 2012 to 2018. Among these cases, 316 (18%) were hospital outbreak-associated cases and 1475 (82%) sporadic cases. The available 120 complete genome sequences (7% of human cases) of MERS-CoV were isolated from 15 cities in Saudi Arabia between 19 June 2012 and 23 January 2017 (Table 1). Of these, 64 (53%) were from hospital outbreak-associated cases, and 56 (47%) were from sporadic cases. The most affected city was Riyadh (55 isolates of 656 cases, 8%), followed by Jeddah (14 isolates of 235 cases, 6%) and Hofuf (12 isolates of 86 cases, 14%). In comparison with the southeast region, a smaller number of MERS-CoV cases and isolates were reported in the northwest region (zero isolates of 19 cases in Tabuk, 21 cases in Hail, eight cases in Arar and 24 cases in Al Jawf). The majority of sequences (*n* = 103, 86%) were matched to individuals in the MERS-CoV human cases epidemiological dataset (Tables S1–S3), including 40 (33%) unique matched isolates and 63 (53%) likely matched isolates. The remaining 17 sequences (14%) were considered as possible matches.

### 3.2. Evolutionary Analysis and Evaluation of Transmission Routes

**Figure 1:**
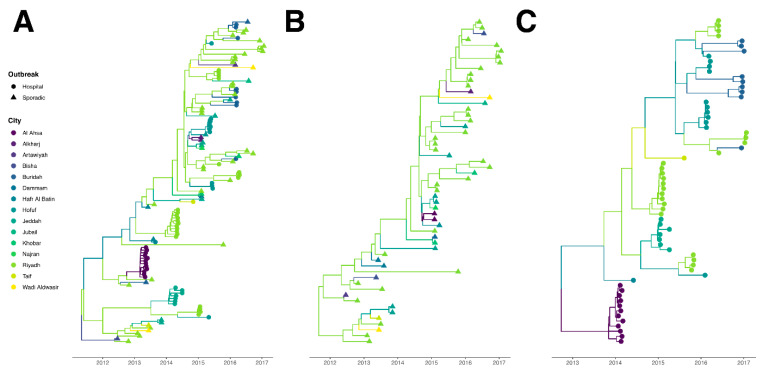
Time-rooted phylogenetic tree of MERS-CoV in Saudi Arabia. Branches are colored by sampling location and tips are shaped by type of case. (**A**) In total, 120 isolates from hospital outbreak-associated cases and sporadic cases. (**B**) In total, 56 isolates from sporadic cases. (**C**) In total, 64 isolates from hospital outbreak-associated cases.

The regression of root-to-tip genetic distances against the sampling year showed clear evidence of clock-like evolution in MERS-CoV (R^2^ = 0.86) (Figure S1). In Figure 1A, we show the time-rooted phylogenetic tree of MERS-CoV over 15 discrete locations in Saudi Arabia. We see the dynamics of MERS-CoV started with sporadic cases, followed by hospital outbreaks. The mean rate of evolution was 7.43 × 10^−4^ (95% HPD interval (6.03 × 10^−4^, 9.16 × 10^−4^)) substitutions per site per year, and the coefficient of variation was 0.83 (95% HPD interval (0.61, 1.06)). The earliest isolate shown in the phylogeny trees was sampled from a sporadic case in Bisha on 19 June 2012, which was consistent with the first human case reported in the literature [36]. Riyadh was the epicenter from 2012 to 2017 with 55 isolates of MERS-CoV, taking 46% of the overall isolates.

**Figure 2:**
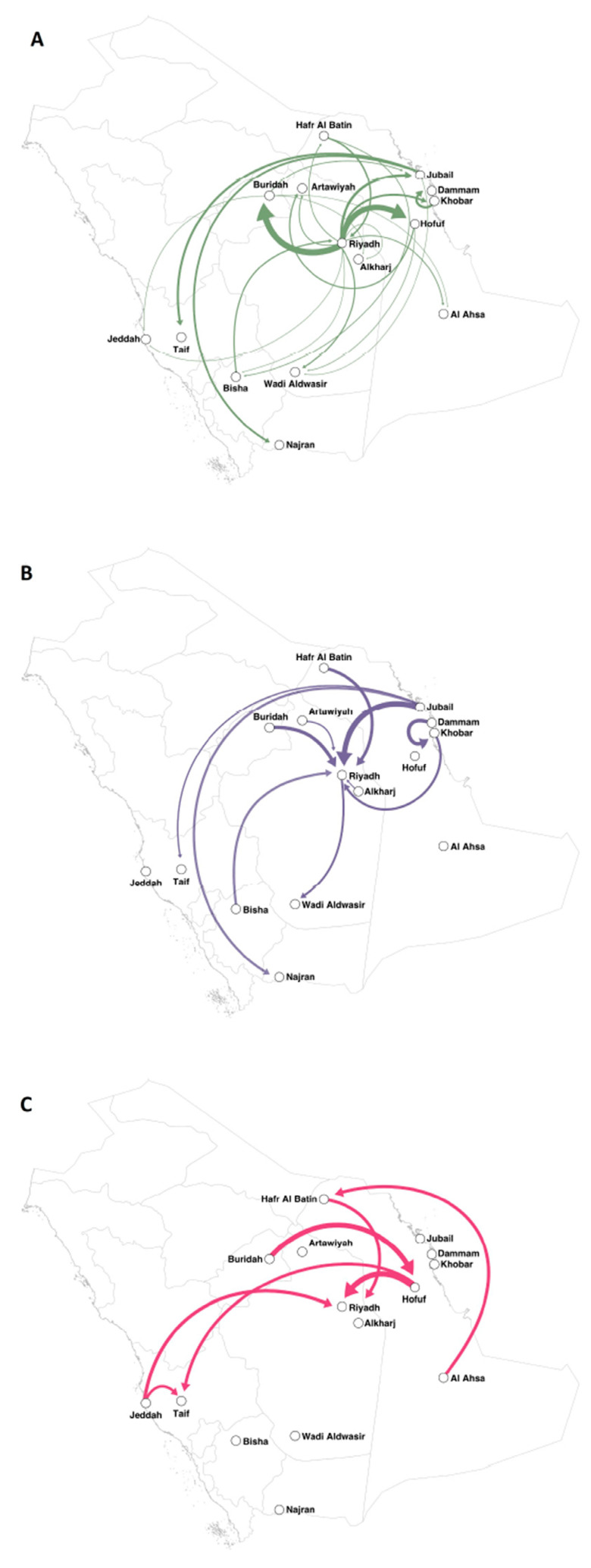
Projection between 15 discrete locations using Bayesian phylogeography. The directions of supportive transmission routes (BF > 3) are indicated by arrows. The thicker arrow indicates the higher BF value. (**A**) Generated from both hospital outbreak-associated cases and sporadic cases. (**B**) Generated from sporadic cases. (**C**) Generated from hospital outbreak-associated cases.

**Figure 3:**
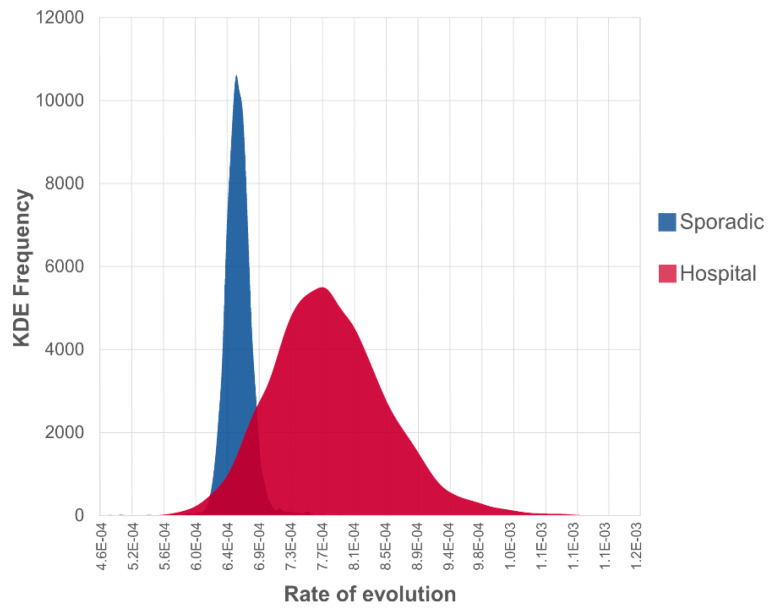
Comparison of Kernel Density Estimate (KDE) of posterior evolutionary rates between hospital outbreak-associated isolates and sporadic community isolates.

We mapped the major cities where MERS-CoV cases were reported and significant supportive transmission routes between 15 discrete locations in Saudi Arabia (Bayes Factor (BF) > 3, Table 2, Figure 2). Based on the epidemic pattern, we have shown the separate phylogenetic trees of isolates from sporadic cases (Figure 1B) and from hospital outbreak-associated cases (Figure 1C). The evolutionary rates are compared between two types of isolates in Figure 3. In the Supplementary Materials, we show three time-rooted phylogenetic trees colored by the evolutionary rate and showing posterior probabilities. The three phylogenetic trees were generated from the overall 120 isolates (Figure S2A): 56 isolates from sporadic cases (Figure S2B) and 64 isolates from hospital outbreak-associated cases (Figure S2C). 

For sporadic cases, isolates were mostly collected in the years 2013, 2015 and 2016 (Figure 1B). A total of 14 discrete locations were involved, of which Riyadh was the hotspot with 32 (57%) isolates recorded. The rate of evolution was 7.58 × 10^−4^ (95% HPD interval (6.10 × 10^−4^, 9.02 × 10^−4^)) substitutions per site per year, and the coefficient of variation was 0.37 (95% HPD interval (0.23, 0.53)). In comparison with hospital-based isolates, the overall evolutionary rate of MERS-CoV was lower in sporadic cases (Figure 3). Of the 56 isolates from sporadic cases, it was found that three isolates sequenced from Riyadh in 2013 and 2017 evolved faster than others (Figure S2C).

Hospital outbreaks occurred between 2013 and 2016, a narrower period than sporadic cases throughout the years between 2012 and 2017. We identified a total of 10 hospital outbreaks in six discrete locations in Saudi Arabia, including eight major outbreaks: (i) Al Ahsa, April–May 2013, (ii) Jeddha, April–July 2013, (iii) Riyadh, April–May 2014, (iv) Taif, November 2014, (v) Hofuf, May–June 2015, (vi) Riyadh, August 2015, (vii) Buraidah, March 2016, (viii) Riyadh, April 2016 and two possible hospital outbreaks: (i) Hafr Al Batin, August 2013 and (ii) Riyadh, January 2015. The estimated evolutionary rate of hospital-based isolates was 1.01 × 10^−3^ (95% HPD interval (7.15 × 10^−4^, 1.38 × 10^−3^)) substitutions per site per year, and the coefficient of variation was 1.18 (95% HPD interval (0.96, 1.42)). The results indicate that in comparison with sporadic cases, viruses from hospital-based cases evolved faster over a shorter time-frame and had the higher variability of sequences. 

For the Al Ahsa hospital outbreak, it was hypothesized that there might be multiple introductions of MERS-CoV strains from the community when using limited sequence data [11]. However, our results showed that the 12 isolates from the Al Ahsa outbreak were monophyletic. We found multiple introductions of sequences in three hospital outbreaks in Hofuf (May–June 2015), Riyadh (August 2015) and Buraidah (March 2016), as the latest ancestors of these MERS-CoV isolates were different clades in each hospital outbreak (Figure 1C). Furthermore, high rates of evolution occurred in MERS-CoV isolates from these three outbreaks (Figure S2B). Based on the sequence data and the matched individuals, we traced the contact history of cases. In the Hofuf hospital outbreak, of the 11 cases which had isolates, only one case had an unknown exposure history. The other 10 cases were exposed to confirmed cases in a hospital, and one of them was also exposed to camels before illness. In the Riyadh hospital outbreak, the index case was exposed to camels before hospitalization, then four cases were infected in the hospital and samplings were collected. In the Buraidah hospital outbreak, all nine isolates were samplings from hospitalized patients, yet the index case is uncertain due to the lack of outbreak information. Complete posterior distribution and likelihood results are included in the supplementary log files accessible at https://figshare.com/s/d6f91d62ada4915fd416.

## 4. Discussion

This study provides further understanding of the dynamics and transmission patterns of MERS-CoV among human cases. The phylogenetic analysis of all the available isolates between 2012 and 2018 showed an evolutionary rate of 7.43 × 10^−4^ (95% HPD interval (6.03 × 10^−4^, 9.16 × 10^−4^)) substitutions per site per year, similar to that (6.3 × 10^−^⁴ substitutions per site per year) when fewer sequences were analyzed in the literature [36]. MERS-CoV is evolving more rapidly than SARS-CoV (4 × 10^−^⁴ substitutions per site per year) [5] and the newly emerged influenza A/H3N2 viruses (0.9–3.1 × 10^−5^ substitutions per site per year) in humans [37], suggesting a decrease in the replication fidelity of MERS-CoV. 

In this study, we linked and used enhanced surveillance datasets for phylogeographic analysis. The MERS-CoV genome sequences were matched with human individuals as well as multiple risk factors. This enabled comparison of the sequences from hospital outbreaks versus sporadic cases, based on the human case contact history. Isolates from hospital outbreaks showed unusual and faster evolutionary speed in a shorter timeframe than sporadic cases. It suggests that MERS-CoV may rapidly mutate in humans in outbreak settings, compared to sporadic cases where human to human transmission is limited. In addition, there was a diversity of ancestral strains in three hospital outbreaks, including the Riyadh outbreak in 2015, the Hofuf outbreak in 2015 and the Buraidah outbreak in 2016. These data suggest multiple introductions of MERS-CoV strains in three separate hospital outbreaks. This phenomenon was first observed in the Al Ahsa hospital outbreak in 2013 [11], in which four MERS-CoV isolates from hospital patients were analyzed and nucleotide differences were found. We enhanced the dataset and identified 12 MERS-CoV isolates from the Al Ahsa hospital outbreak for phylogenetic analysis to minimize the bias caused by the small size of the sequence data. It was found that all of these isolates were related to the same ancestor, although there were slight differences in evolutionary rates between isolates. Thus, these findings showed that the Al Ahsa hospital outbreak in 2013 may have been caused by a single introduction of MERS-CoV from the community.

In Saudi Arabia, although the first isolate of MERS-CoV was from Bisha in 2012 [3], Riyadh was the epicenter of MERS-CoV. This study shows frequent transmissions of viruses between Riyadh and the southeastern cities, and few isolates were available from the northwestern cities due to the lower density of population in the desert areas of the country. 

The primary limitation of this study was sampling bias due to the small number of complete genome sequences of MERS-CoV available from GenBank. This study has covered the largest number of MERS-CoV sequences isolated from humans in Saudi Arabia between 2012 and 2018 [4,36,38,39,40]. Of the overall human cases in Saudi Arabia, however, 93% had no isolates of MERS-CoV. Although some partial genome sequences of MERS-CoV are available in the literature, we excluded them due to insufficient length. Using complete genome sequences of the same length could reduce bias when running phylogenetic analysis. Another limitation of this study was that MERS-CoV genome sequences isolated from animals were not included. As MERS-CoV is considered a zoonotic infectious disease, a phylogeographic analysis using isolates from both humans and animals such as camels would provide further understanding of MERS-CoV dynamics in Saudi Arabia. 

## 5. Conclusions

This study shows the phylogeography of MERS-CoV in Saudi Arabia with a comparison between hospital outbreak-associated cases and sporadic cases using the Bayesian phylogenetic method. MERS-CoV is evolving faster than SARS and even influenza viruses, with a high speed of evolution in hospital outbreaks. The study suggests that MERS-CoV may be rapidly mutating in humans with possible increased transmissibility between humans. The three separate hospital outbreaks with multiple introductions of different strains require further investigation. The use of phylogeography and enhanced surveillance data can be useful to inform future outbreak investigations.

## Figures and Tables

**Table 1:** The number of MERS-CoV complete genome sequences by sampling year and location.

Location	2012	2013	2014	2015	2016	2017	Total
Al Ahsa	0	12	0	0	0	0	12
Alkharj	0	0	0	2	0	0	2
Artawiyah	0	0	0	0	1	0	1
Bisha	1	0	0	0	0	0	1
Buraidah	0	1	0	0	10	0	11
Dammam	0	0	0	1	0	0	1
Hafr Al Batin	0	3	0	0	0	0	3
Hofuf	0	0	0	12	0	0	12
Jeddah	0	2	7	4	1	0	14
Jubail	0	0	0	0	1	0	1
Khobar	0	0	0	1	1	0	2
Najran	0	0	0	1	0	0	1
Riyadh	2	5	11	16	19	2	55
Taif	0	1	1	0	0	0	2
Wadi Aldwasir	0	1	0	0	1	0	2
**Total**	**3**	**25**	**19**	**37**	**34**	**2**	**120**

**Table 2:** Ranked support of transmission routes (BF > 3) between discrete locations.

Origin	Destination	Bayes Factor
Riyadh	Buraidah	119631.3937
Riyadh	Hofuf	29897.87915
Riyadh	Jubail	165.8164344
Khobar	Dammam	109.4201215
Jubail	Taif	81.58842078
Riyadh	Khobar	49.91136624
Jubail	Najran	40.43222377
Hafr Al Batin	Riyadh	21.5996282
Bisha	Riyadh	14.21214846
Hofuf	Artawiyah	12.95700557
Riyadh	Wadi Aldwasir	11.97577204
Riyadh	Artawiyah	9.566752858
Riyadh	Al Ahsa	9.231095454
Buraidah	Jubail	9.159243505
Riyadh	Hafr Al Batin	7.418145007
Hafr Al Batin	Bisha	6.715095512
Hofuf	Wadi Aldwasir	5.413796481
Bisha	Hafr Al Batin	4.648046686
Buraidah	Jeddah	4.456426673
Riyadh	Alkharj	4.401306164
Buraidah	Alkharj	4.00981903
Riyadh	Jeddah	3.994819293
Riyadh	Bisha	3.925163012
Jubail	Wadi Aldwasir	3.721941193
Buraidah	Al Ahsa	3.57086268

## Supplementary Materials

The following are available online at https://www.mdpi.com/1999-4915/12/5/540/s1, Table S1: Criteria in three groups used for matching MERS-CoV complete genome sequences with human cases, Table S2: Matching the unique isolates with individual case based on criteria in Table S1, Table S3.: Compiled data of 120 MERS-CoV complete genome sequences isolated from human sources in Saudi Arabia, 2012–2018, and sequences matched to individual cases, Table S4: Comparison of 16 preliminary models to determine the substitution, molecular clock, and tree prior, Figure S1: Regression of root-to-tip genetic distances of MERS-CoV against year of sampling, Figure S2: Time-rooted phylogenetic trees of MERS-CoV in Saudi Arabia.
