# Supplementary material for: The Phylogeography of MERS-CoV in Hospital Outbreak-Associated Cases Compared to Sporadic Cases in Saudi Arabia
Source: Viruses. 2020 May 14;12(5):540. doi: 10.3390/v12050540 (PMC7290704; doi:10.3390/v12050540)
Supplement: Supplementary file 1 [file viruses-12-00540-s001.pdf]

Supplementary Materials

Table S1. Criteria in three groups used for matching MERS-CoV complete genome sequences with human cases

Group 1. Demographic characteristics	Group 2. Location	Group 3. Date
Age	County or town	Collection date of isolate
Gender	City	Lab confirmation date* (0-10 days after collection date)
Healthcare worker	Province	Symptoms onset date* (14 days before collection date)
Contact history	Region	Death date* (after collection date)

* The closest date from the collection date was selected.

Table S2. Matching the unique isolates with individual case based on criteria in Table S1.

Matching level#	At least one criterion from group
Unique isolate	1 and 2 and 3
Likely unique	2 and 3
Possible unique	2 or 3

Table S3. Compiled data of 120 MERS-CoV complete genome sequences isolated from human sources in Saudi Arabia, 2012-2018, and sequences matched to individual cases.

	Genbank Accession	Date of collection	Location	Type	Matching outcome
1	KF186564	2013-05-01	Al Ahsa	Hospital	Unique
2	KF186565	2013-04-22	Al Ahsa	Hospital	Unique
3	KF186566	2013-04-21	Al Ahsa	Hospital	Unique
4	KF186567	2013-05-09	Al Ahsa	Hospital	Unique
5	KF600612	2012-10-23	Riyadh	Sporadic	Unique
6	KF600613	2013-02-05	Riyadh	Sporadic	Possible
7	KF600620	2012-06-19	Bisha	Sporadic	Unique
8	KF600627	2013-05-07	Al Ahsa	Hospital	Unique
9	KF600628	2013-06-04	Hafr Al Batin	Sporadic	Likely
10	KF600630	2013-05-13	Buridah	Sporadic	Possible
11	KF600632	2013-05-23	Al Ahsa	Hospital	Unique
12	KF600634	2013-05-30	Al Ahsa	Hospital	Unique
13	KF600644	2013-05-12	Al Ahsa	Hospital	Unique
14	KF600645	2013-05-11	Al Ahsa	Hospital	Unique
15	KF600647	2013-05-15	Al Ahsa	Hospital	Unique
16	KF600651	2013-05-23	Al Ahsa	Hospital	Unique
17	KF600652	2012-10-30	Riyadh	Sporadic	Unique
18	KF958702	2013-11-05	Jeddah	Sporadic	Unique
19	KJ156866	2013-05-02	Al Ahsa	Hospital	Unique
20	KJ156869	2013-07-17	Riyadh	sporadic	Likely
21	KJ156874	2013-08-28	Hafr Al Batin	Hospital	Possible
22	KJ156881	2013-06-12	Wadi Aldwasir	Sporadic	Likely
23	KJ156910	2013-08-05	Hafr Al Batin	Sporadic	Likely
24	KJ156934	2013-08-15	Riyadh	Sporadic	Likely
25	KJ156944	2013-07-02	Riyadh	Sporadic	Likely

26	KJ156949	2013-06-12	Taif	Sporadic	Likely
27	KJ156952	2013-03-01	Riyadh	Sporadic	Possible
28	KJ556336	2013-11-06	Jeddah	Sporadic	Unique
29	KM027255	2014-07-01	Jeddah	Hospital	Unique
30	KM027256	2014-07-01	Jeddah	Hospital	Unique
31	KM027257	2014-04-07	Jeddah	Hospital	Unique
32	KM027258	2014-04-12	Jeddah	Hospital	Unique
33	KM027259	2014-04-14	Jeddah	Hospital	Unique
34	KM027260	2014-04-21	Jeddah	Hospital	Unique
35	KM027261	2014-04-15	Jeddah	Hospital	Unique
36	KM027262	2014-04-22	Riyadh	Hospital	Likely
37	KR011263	2015-01-21	Riyadh	Hospital	Likely
38	KR011264	2015-01-21	Riyadh	Hospital	Likely
39	KR011265	2015-01-26	Riyadh	Hospital	Likely
40	KR011266	2015-01-06	Riyadh	Hospital	Likely
41	KT026453	2015-02-10	Riyadh	Sporadic	Likely
42	KT026454	2015-03-01	Riyadh	Sporadic	Likely
43	KT026455	2015-02-10	Riyadh	Sporadic	Likely
44	KT026456	2015-03-01	Riyadh	Sporadic	Likely
45	KT121572	2014-05-12	Riyadh	Hospital	Unique
46	KT121573	2014-05-09	Riyadh	Hospital	Unique
47	KT121574	2014-05-07	Riyadh	Hospital	Unique
48	KT121575	2014-05-12	Riyadh	Hospital	Unique
49	KT121576	2014-05-18	Riyadh	Hospital	Unique
50	KT121577	2014-05-11	Riyadh	Hospital	Unique
51	KT121578	2014-05-01	Riyadh	Hospital	Unique
52	KT121579	2014-04-30	Riyadh	Hospital	Unique
53	KT121580	2014-04-28	Riyadh	Hospital	Unique
54	KT121581	2014-05-03	Riyadh	Hospital	Unique
55	KT806044	2015-02-09	Jeddah	Sporadic	Likely
56	KT806045	2015-02-22	Jeddah	Sporadic	Likely
57	KT806046	2015-05-10	Hofuf	Hospital	Likely
58	KT806047	2015-03-27	Hofuf	Sporadic	Likely
59	KT806048	2015-02-07	Dammam	Sporadic	Likely
60	KT806049	2015-02-15	Riyadh	Sporadic	Likely
61	KT806051	2015-02-05	Riyadh	Sporadic	Likely
62	KT806052	2015-02-02	Alkharj	Sporadic	Likely
63	KT806053	2015-02-02	Alkharj	Sporadic	Likely
64	KT806054	2015-02-13	Najran	Sporadic	Likely
65	KT806055	2015-02-10	Jeddah	Sporadic	Likely
66	KU710264	2014-11-04	Taif	Hospital	Unique
67	KU851859	2015-07-12	Jeddah	Sporadic	Possible
68	KU851860	2015-08-24	Riyadh	Hospital	Unique
69	KU851861	2015-08-24	Riyadh	Hospital	Unique

70	KU851862	2015-08-24	Riyadh	Hospital	Unique
71	KU851863	2015-08-27	Riyadh	Hospital	Unique
72	KU851864	2015-08-24	Riyadh	Hospital	Unique
73	KX154684	2016-02-01	Riyadh	Sporadic	Possible
74	KX154685	2016-02-01	Riyadh	Sporadic	Possible
75	KX154686	2016-02-01	Riyadh	Sporadic	Possible
76	KX154687	2016-02-01	Riyadh	Sporadic	Possible
77	KX154688	2016-02-01	Riyadh	Sporadic	Possible
78	KX154689	2016-01-01	Riyadh	Sporadic	Possible
79	KX154690	2016-01-01	Jeddah	Sporadic	Possible
80	KX154691	2016-02-25	Riyadh	Sporadic	Likely
81	KX154692	2016-02-27	Riyadh	Sporadic	Possible
82	KX154693	2016-02-29	Riyadh	Sporadic	Likely
83	KX154694	2016-02-29	Artawiyah	Sporadic	Likely
84	KY688118	2015-02-07	Khobar	Sporadic	Possible
85	KY688119	2015-05-01	Hofuf	Hospital	Likely
86	KY688120	2015-05-10	Hofuf	Hospital	Likely
87	KY688121	2015-05-22	Hofuf	Hospital	Likely
88	KY688122	2015-05-27	Hofuf	Hospital	Likely
89	KY688123	2015-05-13	Hofuf	Hospital	Likely
90	KY688124	2015-05-20	Hofuf	Hospital	Likely
91	MG011340	2016-03-05	Buridah	Hospital	Likely
92	MG011341	2016-03-06	Buridah	Hospital	Likely
93	MG011342	2016-03-07	Buridah	Hospital	Likely
94	MG011343	2016-03-12	Buridah	Hospital	Likely
95	MG011344	2016-03-13	Buridah	Hospital	Likely
96	MG011345	2016-03-18	Buridah	Hospital	Likely
97	MG011346	2016-03-14	Buridah	Hospital	Likely
98	MG011347	2016-03-13	Buridah	Hospital	Likely
99	MG011348	2016-04-18	Riyadh	Hospital	Likely
100	MG011349	2016-04-13	Riyadh	Hospital	Likely
101	MG011350	2016-04-04	Riyadh	Hospital	Possible
102	MG011351	2016-04-10	Khobar	Sporadic	Likely
103	MG011352	2016-07-20	Buridah	Sporadic	Likely
104	MG011353	2016-06-01	Riyadh	Sporadic	Likely
105	MG011354	2016-06-16	Riyadh	Sporadic	Likely
106	MG011355	2016-07-01	Riyadh	Sporadic	Possible
107	MG011356	2016-07-12	Riyadh	Sporadic	Possible
108	MG011357	2016-09-21	Riyadh	Sporadic	Likely
109	MG011358	2016-09-21	Wadi Aldwasir	Sporadic	Likely
110	MG011359	2016-03-29	Buridah	Hospital	Likely
111	MG011360	2017-01-23	Riyadh	Sporadic	Likely
112	MG011361	2017-01-10	Riyadh	Sporadic	Likely
113	MG011362	2016-12-25	Riyadh	Sporadic	Likely

114	MG366880	2015-06-02	Hofuf	Hospital	Likely
115	MG366881	2015-06-07	Hofuf	Hospital	Likely
116	MG366882	2015-06-07	Hofuf	Hospital	Likely
117	MG366883	2015-06-15	Hofuf	Hospital	Likely
118	MH013216	2015-10-15	Riyadh	Sporadic	Likely
119	MH306207	2016-08-01	Jubail	Sporadic	Likely
120	MH454272	2016-12-06	Riyadh	Sporadic	Likely

Table S4. Comparison of 16 preliminary models to determine the substitution, molecular clock, and tree prior.

	GTR				HKY			
	Relaxed		Strict		Relaxed		Strict	
	PS	SS	PS	SS	PS	SS	PS	SS
Exponential	-51802.0112	-51802.40565	-51910.80245	-51911.0769	-51973.31591	-51974.89481	-52034.80438	-52036.00526
Skyrgid	-51846.35844	-51846.02546	-51904.99512	-51905.46431	-51971.15772	-51972.29083	-52029.37891	-52031.00919
Skyline	-51846.94789	-51848.63296	-51906.30971	-51907.50045	-51977.32287	-51978.71641	-52037.35684	-52038.0191
Constant	**-47635.33113**	**-47635.91342**	-51917.0388	-51917.67271	-51980.52668	-51981.33427	-52047.69012	-52048.45935

Figure S1. Regression of root-to-tip genetic distances of MERS-CoV against year of sampling*

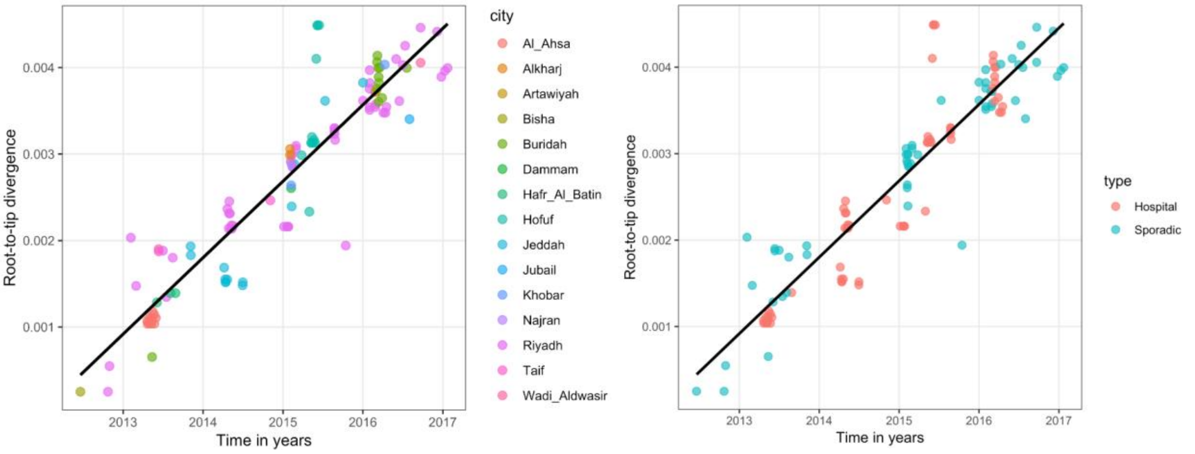

** y=8.8376E-4x+2011.9608*

Figure S2. Time-rooted phylogenetic trees of MERS-CoV in Saudi Arabia. Edges are coloured by evolutionary rate. Values on ancestral nodes represent posterior probabilities. **A.**120 isolates from hospital outbreak-associated and sporadic cases. **B.** 56 isolates from sporadic cases. **C**. 64 isolates from hospital outbreak-associated cases.

A

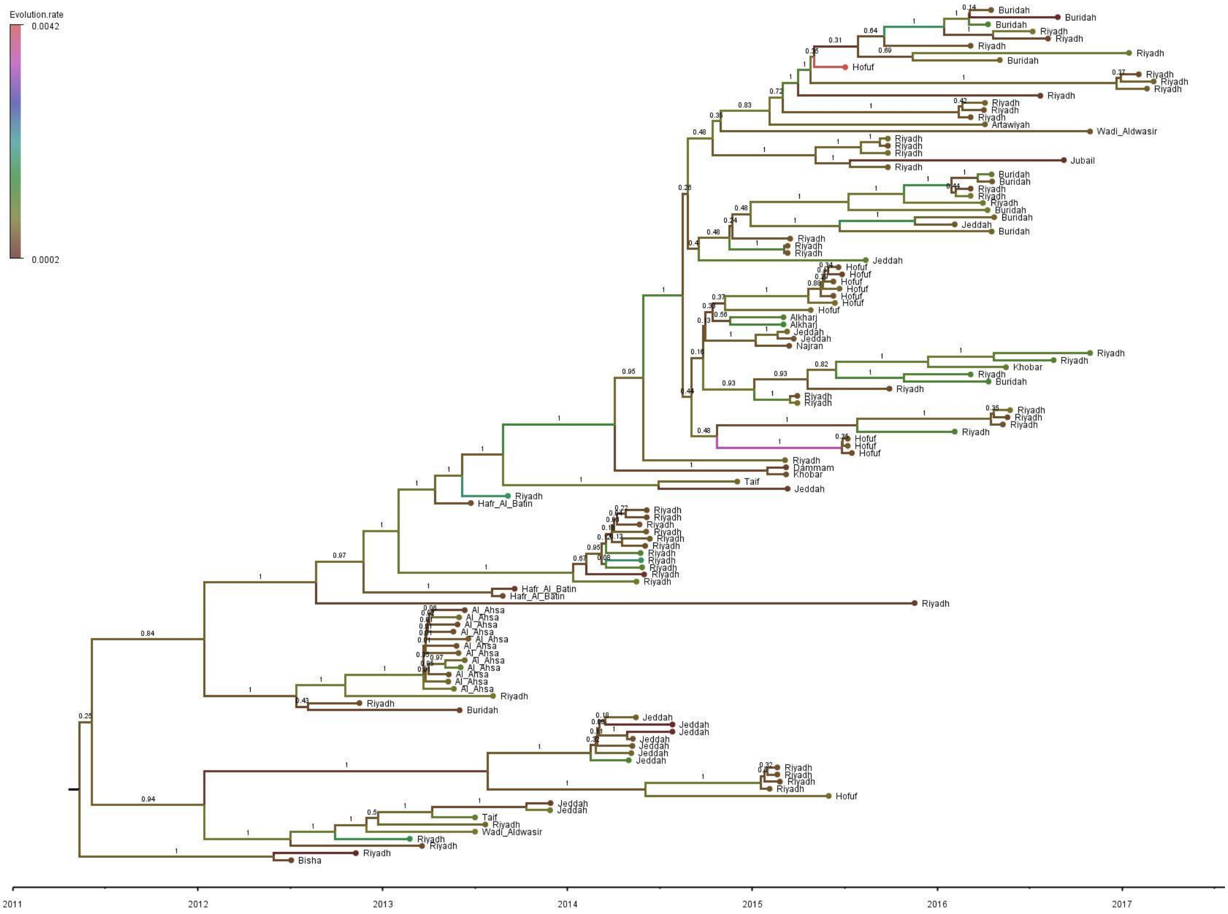

B

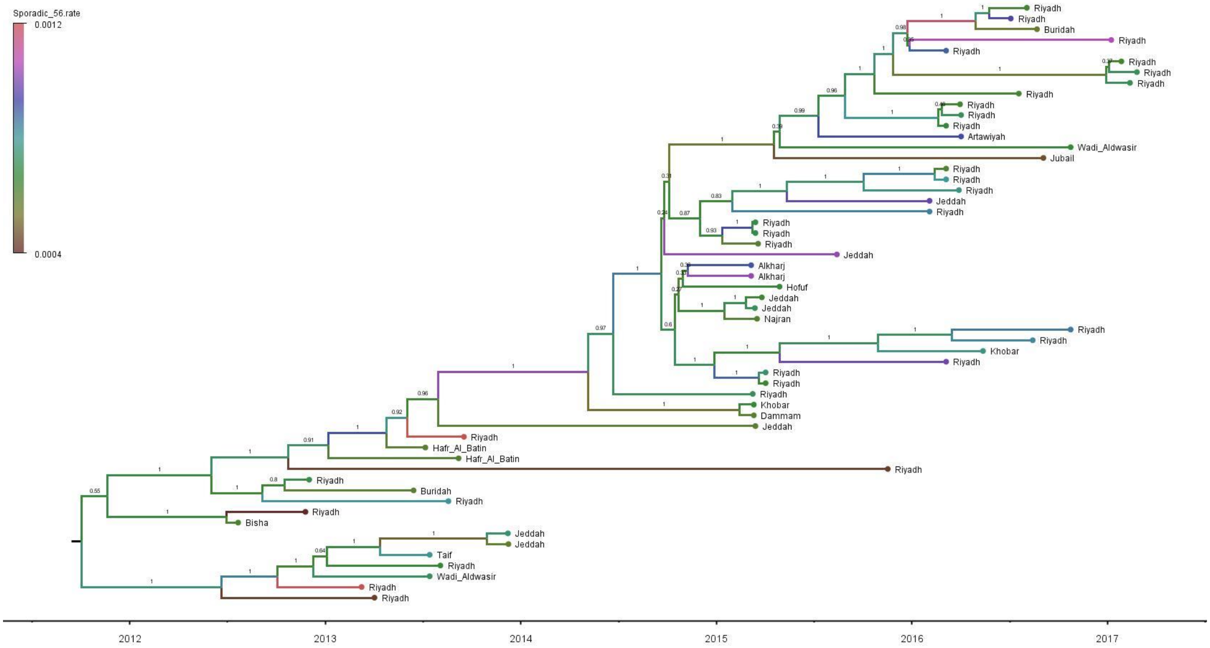

C

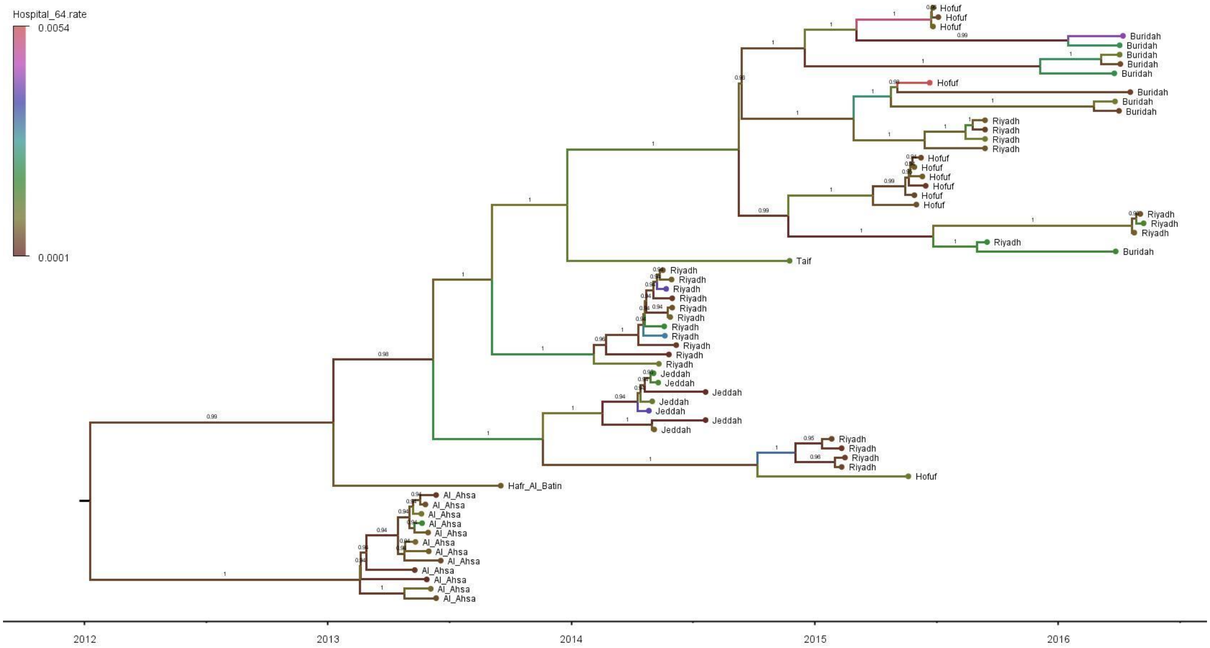

Note: The complete model specification including prior parameter distributions is included in the supplementary file MERS120_CityBSSVS_TypeBSSVS_50M.xml via https://figshare.com/s/64f8f4dbdbcaaa4a8af4. And the complete posterior distribution and likelihood results are included in the supplementary log files via https://figshare.com/s/d6f91d62ada4915fd416.
